# Unfolded Protein Response Suppression in Yeast by Loss of tRNA Modifications

**DOI:** 10.3390/genes9110516

**Published:** 2018-10-23

**Authors:** Alexander Bruch, Roland Klassen, Raffael Schaffrath

**Affiliations:** Institut für Biologie, Fachgebiet Mikrobiologie, Universität Kassel, Heinrich-Plett-Str. 40, D-34132 Kassel, Germany; alexander-bruch@student.uni-kassel.de (A.B.); roland.klassen@uni-kassel.de (R.K.)

**Keywords:** tRNA anticodon modifications, unfolded protein response, tunicamycin, yeast, elongator complex, Deg1

## Abstract

Modifications in the anticodon loop of transfer RNAs (tRNAs) have been shown to ensure optimal codon translation rates and prevent protein homeostasis defects that arise in response to translational pausing. Consequently, several yeast mutants lacking important anticodon loop modifications were shown to accumulate protein aggregates. Here we analyze whether this includes the activation of the unfolded protein response (UPR), which is commonly triggered by protein aggregation within the endoplasmic reticulum (ER). We demonstrate that two different aggregation prone tRNA modification mutants (*elp6 ncs2*; *elp3 deg1*) lacking combinations of 5-methoxycarbonylmethyl-2-thiouridine (mcm^5^s^2^U: *elp3*; *elp6*; *ncs2*) and pseudouridine (Ψ: *deg1*) reduce, rather than increase, splicing of *HAC1* mRNA, an event normally occurring as a precondition of UPR induction. In addition, tunicamycin (TM) induced *HAC1* splicing is strongly impaired in the *elp3 deg1* mutant. Strikingly, this mutant displays UPR independent resistance against TM, a phenotype we found to be rescued by overexpression of tRNA^Gln^(UUG), the tRNA species usually carrying the mcm^5^s^2^U34 and Ψ38 modifications. Our data indicate that proper tRNA anticodon loop modifications promote rather than impair UPR activation and reveal that protein synthesis and homeostasis defects in their absence do not routinely result in UPR induction but may relieve endogenous ER stress.

## 1. Introduction

Transfer RNA (tRNA) is extensively modified to fine-tune the efficiency of translation. Various tRNA modifications are known to contribute to the maintenance of optimal codon translation rates in yeast by preventing ribosomal pausing during the decoding process [1,2]. For example, 5-methoxycarbonylmethyl-2-thiouridine (mcm^5^s^2^U) is present at the wobble position in tRNA^Gln^(UUG) and tRNA^Lys^(UUU) and is required for efficient translation of the cognate A-ending codons by both tRNAs [2,3,4]. Formation of the mcm^5^s^2^U modification requires the Elongator complex and regulatory proteins, as well as Trm9/Trm112 methyl-transferase [5,6,7,8,9]. The thiomodification is separately installed via a sulfur transfer pathway involving Nfs1, Tum1, Uba4, Urm1 and thiolase Ncs2-Ncs6 [10,11,12]. In budding yeast, wobble uridine thiolation and mcm^5^U modification occur partially independent of each other. Loss of each part (mcm^5^U or s^2^U) alone induces shared pleiotropic phenotypes which are aggravated upon combined loss of both [2,13,14,15,16]. Similarly, ribosomal pausing is increased in double mutants lacking mcm^5^U in tandem with s^2^U (*ncs2 elp6*), which goes along with a severe protein homeostasis defect including the accumulation of protein aggregates [2].

In mice, loss of the mcm^5^ moiety alone was shown to induce ribosomal pausing and trigger the unfolded protein response (UPR) within the endoplasmic reticulum (ER), which is assumed to contribute to associated defects in neurodevelopment causing microcephaly [17]. In humans, various mutations in Elongator subunit genes are correlated with different pathologies involving neurodegeneration [18,19], pointing to a positive role of the Elongator in neurodevelopment and in protection against neurodegeneration. Since protein misfolding plays a key role in various neurodegenerative diseases and proteopathies [20], maintenance of translational efficiency and protein homeostasis might represent a functional link between tRNA modification and neurogenesis. 

Another tRNA modification recently linked to human neuropathies is Deg1/Pus3 dependent formation of pseudouridine (Ψ38/39), since homozygous *DEG1/PUS3* mutations are linked to a severe form of intellectual disability [21], a syndrome which can also be caused by human Elongator defects [22]. In yeast, a combination of Elongator and *deg1* mutations was shown to cause severe growth impairment and was associated with the formation of protein aggregates [16]. Furthermore, defects in Deg1 or Elongator dependent modification and tRNA thiolation copy pleiotropic phenotypes in yeast, including temperature and rapamycin sensitivity [3,13,23,24], which is consistent with shared or related cellular roles for these tRNA modifications. For the *elp3 deg1* mutant, severe translational inefficiency and growth phenotypes were shown to be suppressible by elevated copy numbers of tRNA^Gln^(UUG), suggesting this tRNA critically relies on the joint presence of mcm^5^s^2^U and Ψ38 [16,25]. Hence, both anticodon loop modifications appear to collaborate in tRNA^Gln^(UUG) functioning and translational efficiency [16,19].

Since protein aggregation may represent an important pathomechanism of tRNA modification defects in humans, a further characterization of tRNA linked protein homeostasis defects in the yeast model system appears justified. In this study, we aimed to clarify whether combined tRNA modification defects resulting in protein aggregation in yeast occur concomitantly with an induction of the UPR system as observed in the mice model system. Onset of UPR involves detection of ER-based protein homeostasis defects via the endonuclease Ire1, which mediates unconventional splicing of the *HAC1* mRNA [26,27,28,29]. Only after *HAC1* splicing is the functional Hac1 transcription factor produced, which is subsequently involved in the induction of ER localized chaperones to restore ER protein homeostasis [29].

Here, we demonstrate that two different combined tRNA modification defects known to result in protein aggregation in yeast, do so without concomitant induction of UPR. Moreover, basal UPR induction levels are decreased rather than increased, and genetic evidence points to the relief of basal ER stress levels due to severe translational defects as observed in the composite *elp3 deg1* tRNA modification mutant. Hence, yeast models might differ considerably from metazoan neuronal cells in their cellular responses upon interference with tRNA anticodon modifications and inappropriate decoding.

## 2. Materials and Methods

### 2.1. Strains, Plasmids and Cultivation

The strains used and generated in this study are listed in Table 1. Genomic deletions were conducted by PCR mediated protocols based on the pUG plasmid set [30] with oligonucleotides listed in Table 2. Gene replacements were verified with forward/reverse primers positioned outside of the target loci (Table 2). Cultivation of the different strains with yeast nitrogen base (YNB)/yeast peptone dextrose (YPD) was performed using standard methods [31]. Overexpression of the tRNA^Gln^(UUG) utilized pRK55, a YEplac195-based construct [16] and an empty vector served as a control. The designated strains were transformed as previously published [32].

### 2.2. Liquid Growth Inhibition Assays

The mutants as well as the transformants used in this study were adjusted to OD_600_ = 0.025 and grown in a 96-well plate for 24 h in YNB or YPD media with serially increasing concentrations of tunicamycin (TM) used in every well. For each strain, three independent cultures were incubated at the above-mentioned conditions. After the incubation, optical density (OD_600_) was measured using an Epoch Micro–Volume Spectrophotometer System (BioTek, Winooski, VT, USA). Relative growth of the different yeast strains was calculated by normalizing the optical densities with TM against those without.

### 2.3. Isolation of Total RNA

Yeast cultures were incubated until OD_600_ = 1.0 was reached, and total RNA was isolated using the RNeasy Mini Kit (Qiagen, Hilden, Germany) following the instructions of the manufacturer. The isolated RNA was then used for qRT-PCR or RT-PCR experiments.

### 2.4. Quantification of mRNA Levels by qRT-PCR

As a control of UPR induction, splicing of the *HAC1* mRNA in the indicated strains was induced in cultures after OD_600_ = 1.0 was reached, by the addition of 0.5 µg/mL TM for 3 h. To quantify *HAC1^i^* mRNA levels from three biological replicates, the SensiFAST SYBRR^®^ No-ROX Kit (Bioline, Luckenwalde, Germany) and Mastercycler ep realplex (Eppendorf, Hamburg, Germany) were used, applying technical triplicates for each sample, following the manufacturers’ protocols and primers listed in Table 2. Calculation of the relative mRNA level of *HAC1^i^* in the different strains and under various conditions was achieved by normalizing against the quantified amount of *ACT1* mRNA according to [33] whereas a two-tailed *t*-test was used for statistical analyses.

### 2.5. RT-PCR of HAC1 mRNA

For the cDNA synthesis, the RevertAid First Strand cDNA Synthesis Kit (Thermo Fisher, Waltham, MA, USA) was used applying 1 µg of total RNA. The synthesized cDNA was conducted for PCR following established procedures [34] and using the primer HAC1F/R or qPCR_ACT1_FW/RV (PCR-protocol add-on: annealing temperature 60 °C for the control (Table 2), respectively. To separate the PCR products a 2% agarose gel was used. The expected RT-PCR product sizes of unspliced and spliced *HAC1* mRNA are 720 bp and 470 bp, or 168 bp for the *ACT1* mRNA, respectively [34,35].

## 3. Results and Discussion

### 3.1. Dysfunction of the UPR System in tRNA Modification Mutants

Loss of different critical tRNA modifications in yeast induces accumulation of protein aggregates [2,16,36]. However, it has remained unknown whether protein aggregation induced by tRNA defects occurs solely in the cytoplasm, or also within the ER. Importantly, protein aggregation in the ER is triggered in mice and flies in response to tRNA defects, and activates a transcriptomic change termed the unfolded protein response (UPR) [17]. UPR is mediated by the transcription factor Hac1 and its activation requires splicing of the *HAC1* mRNA [26,28,29,37]. To test whether the UPR system is induced in yeast tRNA modification mutants lacking mcm^5^U or s^2^U in combination or together with Ψ38/39, we used RT-PCR to detect *HAC1* mRNA splicing. This Ire1 dependent processing step is a prerequisite for UPR induction in yeast [26,28,29].

**Figure 1:**
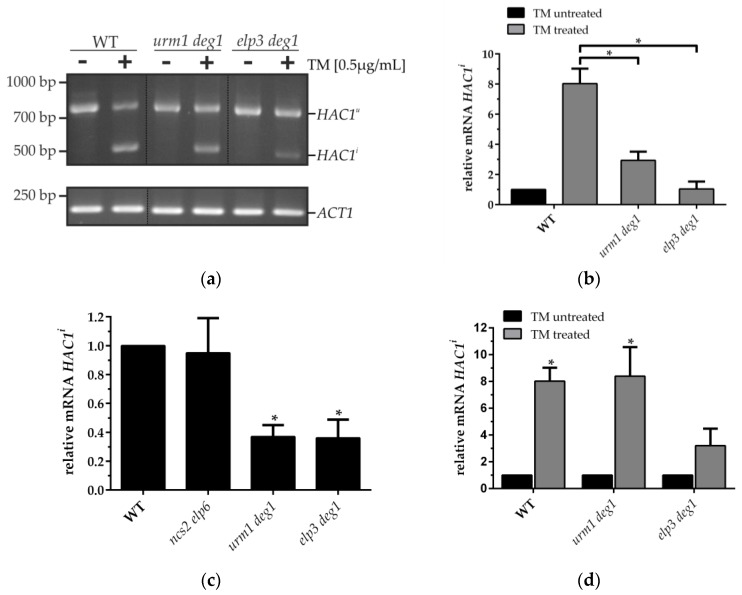
Analysis of basal and tunicamycin (TM) induced *HAC1* splicing in various transfer RNA (tRNA) modification mutants. (**a**) To measure the splicing of *HAC1* mRNA in wildtype (WT), *elp3 deg1* and *urm1 deg1* cells RT-PCR was conducted as described [34]. In each sample, *ACT1* mRNA was detected as a control. Yeast strains were cultivated in yeast peptone dextrose (YPD) until OD_600_ = 1.0 and harvested for RNA extraction. As a control, WT was additionally treated with TM (0.5 µg/mL) for 3 h (+). *HAC1^u^* represents the unspliced *HAC1* mRNA and *HAC1^i^* the mature spliced *HAC1* mRNA. (**b**–**d**) Quantification of the *HAC1^i^* mRNA level without (**b**), and with TM treatment (**c**,**d**) via qRT-PCR of the indicated strains. Induction of *HAC1* splicing with TM was carried out as described in (**a**). mRNA levels were normalized to *ACT1* using the ΔΔCt method [33]. The results obtained with TM treated yeast strains were standardized against the *HAC1^i^* mRNA level of the untreated wild-type (**c**) or the corresponding untreated strains (**d**), respectively. Quantitative PCR was performed with at least three biological triplicates per strain and condition and statistical significance was determined using a two-tailed *t*-test and indicated in the bar charts (* *p* < 0.05, **b**–**d**).

As seen in Figure 1a, the wild type (WT) and double tRNA modification mutants contained only unspliced *HAC1^u^* mRNA under optimal growth conditions basedon detection by the RT-PCR assay [34]. However, when WT cells were treated with TM, a well-known inhibitor of *N*-linked glycosylation [38] and therefore an UPR inducer in yeast, RT-PCR-based detection of spliced *HAC1* mRNA was facilitated. Thus, absence of the tested tRNA modifications does not appear to induce splicing of *HAC1* mRNA in a fashion, and to the extent, comparable to TM. To check for minor induction of *HAC1* splicing in the tRNA modification mutants which may have escaped detection by the semi-quantitative RT-PCR assay, we devised a highly sensitive qRT-PCR based strategy to quantify spliced *HAC1* mRNA (*HAC1^i^*). This strategy involves a set of oligonucleotides (Table 2), which generate a product only from spliced *HAC1* mRNA. Indeed, TM exposure of WT cells resulted in an ~8-fold induction of spliced *HAC1* levels, demonstrating the suitability of this assay to monitor UPR induction in yeast (Figure 1c).

When applying the quantitative assay to RNA from TM untreated tRNA modification mutants *urm1 deg1* (s^2^U and Ψ38/39 deficient), *elp3 deg1* (mcm^5^U and Ψ38/39 deficient) or *ncs2 elp6* (mcm^5^s^2^U deficient) no elevated *HAC1* splicing levels compared to the WT were observed (Figure 1b). In contrast, *urm1 deg1* and *elp3 deg1* mutants displayed significantly reduced *HAC1* splicing levels, while the *ncs2 elp6* mutant and the WT contained similar amounts of *HAC1^i^* (Figure 1b). Together these results indicate that observed protein homeostasis defects in *ncs2 elp6* and *elp3 deg1* [2,16] appear not to be associated with the induction of the UPR system. Hence, protein aggregation either does not include ER proteins, or is not sufficient to activate *HAC1* splicing and subsequent transcriptional changes mediated by Hac1. In support of this notion, previous RNAseq data did not indicate an upregulation of UPR genes in the *ncs2 elp6* mutant, and among the identified proteins in aggregates, cytosolic ones were strongly enriched whereas ER proteins were under-represented (Table S1) [2].

Since absence of UPR induction in aggregation prone tRNA modification mutants might be explainable either with absence of ER resident protein aggregates or with a UPR functional defect, we tested whether the UPR system is still functional in *urm1 deg1* and *elp3 deg1* mutants. We used TM to induce the *HAC1* mRNA splicing [38], which we monitored using the RT-PCR approach. The WT showed after treatment with TM both spliced and unspliced *HAC1* variants, respectively (Figure 1a, lane 2). The double mutants also displayed both *HAC1^u^* and *HAC1^i^* mRNAs following TM exposure (Figure 1a, lanes 3-6), indicating the functionality of the UPR system in these mutants. Interestingly, by comparing the intensity of the slower and faster migrating bands in *urm1 deg1* mutant cells with the ones of the WT, it seemed that the *HAC1^i^* mRNA level was reduced in that mutant (Figure 1a, lanes 2 and 4). This effect was stronger for *elp3 deg1* (Figure 1a, lanes 2 and 6).

To compare splicing efficiency in WT and tRNA modification mutant cells after TM exposure, we used qRT-PCR-based quantification of spliced *HAC1* mRNA. Indeed, the double mutants showed a strong reduction in the amount of the TM-induced *HAC1* splice product (Figure 1c, lanes 3 and 4). While TM treatment of WT cells induced *HAC1^i^* formation ~8-fold, this was reduced in *urm1 deg1* (~3-fold) and *elp3 deg1* (no induction), when standardized to the *HAC1^i^* level of the untreated WT, respectively. To further test if the double mutants additionally showed a reduced splicing rate of *HAC1* mRNA, we set the *HAC1^i^* level of untreated strains as a standard and compared each to the amount after treatment, respectively (Figure 1d). While WT and *urm1 deg1* displayed similar relative changes (~8-fold), the *elp3 deg1* mutant showed decreased *HAC1^i^* inducibility (~3-fold) pointing to a reduced capacity to activate UPR.

Taken together, the tRNA modification double mutants are still capable of induced *HAC1* mRNA splicing, but absolute *HAC1^i^* levels are reduced compared to the WT. Based on earlier studies, which revealed an accumulation of misfolded proteins in *ncs2 elp6* and *elp3 deg1* double mutants [2,16], it seems counterintuitive that these mutants exhibit reduced basal UPR activation and reduced capacity to initiate UPR upon exogenously induced ER stress. Possibly, protein aggregation in combined tRNA modification mutants is limited to the cytoplasm and reduced amounts of misfolded proteins are present in the ER. In support of this notion, it has been shown for the mcm^5^s^2^U deficient mutant *ncs2 elp6* that only a small amount of the protein aggregates formed are ER-related [2].

### 3.2. Rescue of UPR Suppression by tRNA Overexpression

The above results revealed reduced *HAC1^i^* levels in combined tRNA modification mutants both before and after TM exposure, consistent with a general UPR defect. In several cases, downstream cellular effects resulting from translational defects and loss of critical tRNA anticodon loop modifications have been shown to be suppressed by overexpression of the tRNA substrates usually carrying the appropriate modifications [3,13,16,25,39,40]. Importantly, translational defects and phenotypes of the *elp3 deg1* mutant were already shown to be suppressible by overexpression of tRNA^Gln^(UUG), the single tRNA in yeast normally carrying the mcm^5^s^2^U and Ψ38 modifications in the anticodon stem loop [16]. Here we determined whether UPR defects of this mutant are similarly suppressible by tRNA overexpression. If so, UPR defects would likely occur as an indirect consequence of translational inefficiency, similar to the majority of pleiotropic phenotypes observed in Elongator and Deg1 deficient (*elp3 deg1*) cells.

**Figure 2:**
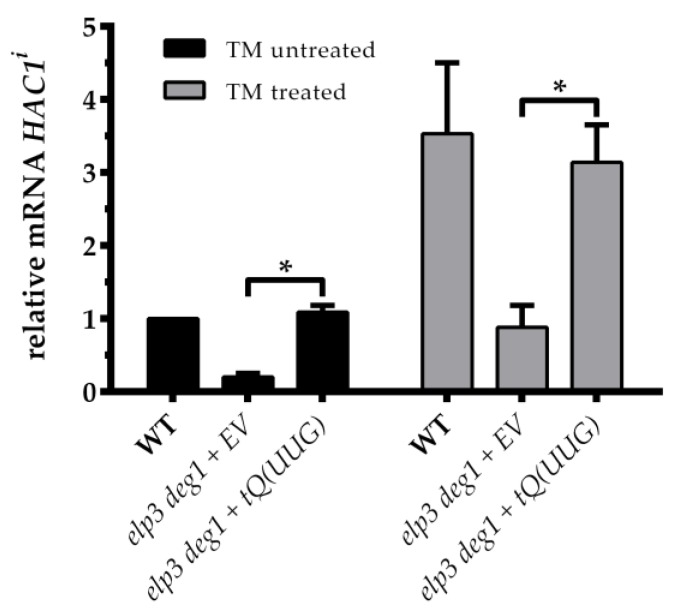
Overexpression of tRNA^Gln^(UUG) reverts the *HAC1* mRNA splicing phenotype of the modification mutants. The indicated strains carrying either the empty vector (EV) or tRNA^Gln^(UUG) (*tQUUG*) expressing vector were grown in YNB until OD_600_ = 1.0 before RNA extraction. *HAC1^i^* mRNA levels (before/after TM treatment) of the indicated transformants were quantified. Statistical significance was determined using a two-tailed *t*-test (* *p* < 0.05).

We thus first measured basal *HAC1* splicing in *elp3 deg1* mutant cells carrying either the empty vector or the tRNA^Gln^(UUG) overexpression vector that had previously been shown to rescue multiple phenotypes of the double mutant. We found that the presence of the tRNA^Gln^(UUG) expression vector resulted in a robust ~3-fold increase in basal *HAC1* splicing in relation to the empty vector control (Figure 2). Basal *HAC1* splicing levels were in fact restored to WT levels (Figure 2). Next, we analyzed whether tRNA^Gln^(UUG) overexpression also suppresses the UPR induction defect of the double mutant after exposure to TM. Again, *HAC1* splicing after TM treatment was significantly higher in response to the tRNA^Gln^(UUG) overexpression construct compared to the empty vector control. Moreover, TM induced *HAC1*^i^ levels of the *elp3 deg1* mutant were restored nearly to WT levels in the presence of the tRNA^Gln^(UUG) overexpression vector (Figure 2). Together, our results suggest that the observed UPR defect in the combined tRNA modification mutants highly likely lies with an indirect consequence of translational inefficiency, since it is suppressible by overexpression of the hypomodified tRNA^Gln^(UUG), which causes the translational defect in the first place.

### 3.3. TM Phenotypes of tRNA Modification Mutants and Their Response to tRNA^Gln^(UUG) Overexpression

**Figure 3:**
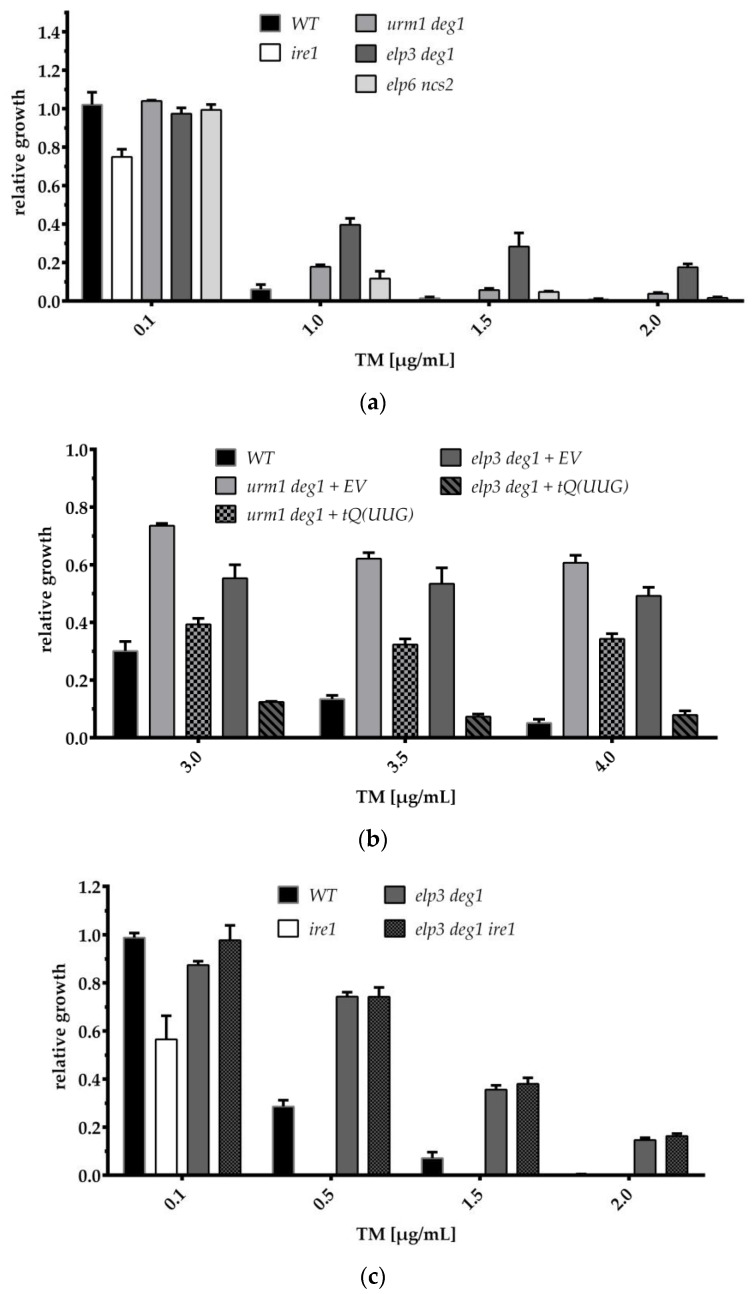
TM phenotypes of tRNA modification mutants and their response to tRNA^Gln^(UUG) overexpression. The indicated yeast strains were cultivated in YPD (**a**,**c**) or in YNB (**b**) 24 h with indicated concentrations of TM. Each experiment involved three biological replicates, and the resulting standard deviations are indicated on the bars. EV: empty vector; *tQUUG*: tRNA^Gln^(UUG) expressing vector.

It has been demonstrated in different studies that the inability to induce the UPR system or downstream proteins results in a strong sensitivity against ER stress-inducing agents such as TM [41,42,43]. To test whether the tRNA modification mutants display altered TM sensitivity due to the reduced UPR induction we conducted liquid growth inhibition assays. At a TM concentration of 0.1 µg/mL, both the WT and the tRNA modification mutants (*urm1 deg1*; *elp3 deg1*; *ncs2 elp6*) showed a relative growth rate of about 100% (Figure 3a). At higher TM doses, the WT undergoes stepwise growth reduction, while *urm1 deg1*, *elp3 deg1* and *ncs2 elp6* mutants displayed partial TM resistance towards the ER stress-inducing agent. These phenotypes were unexpected concerning the strongly reduced *HAC1^i^* mRNA levels in the first two modification mutants (Figure 1c). Interestingly however, absence of the s^2^U part (of the mcm^5^s^2^U modification) in several Urm1 pathway mutants was already shown to confer resistance to ER stress induced by TM as well [44]. Moreover, it was demonstrated that overexpression of three tRNAs naturally carrying the s^2^U modification was sufficient to restore normal sensitivity to TM in a s^2^U deficient mutant [44]. Our observation that combined loss of tRNA modifications (including and excluding s^2^U) causes a *HAC1* maturation defect and TM resistance, suggested a more general functional link between translational defects induced by inappropriate tRNA modifications and resistance to ER stress. However, it remained to be determined whether both, UPR induction and resistance to TM induced ER stress are jointly suppressible by tRNA overexpression. To test this, we compared TM resistance of *urm1 deg1* and *elp3 deg1* double mutants in the presence of either the empty vector or the tRNA^Gln^(UUG) overexpression construct.

As shown in Figure 3b, at TM concentrations between 3 and 4 µg/mL, the presence of the tRNA^Gln^(UUG) vector correlated with significantly reduced growth as compared to the empty vector control, for both *urm1 deg1* and *elp3 deg1* double mutants. Hence, tRNA^Gln^(UUG) overexpression does not only restore *HAC1* splicing in the absence and presence of TM (Figure 2), but also restores TM sensitivity of mutant strains. We further observed that the TM response differed depending on whether yeast cells were cultivated in rich or minimal medium with higher degrees of resistance observable in the latter condition (Figure 3a,b).

Interestingly, deletion of different ribosomal protein (RP) genes was shown to induce a similar TM resistance in yeast, and the strength of the TM effect correlated with the strength of growth inhibition due to loss of the individual RP gene [45]. Thus, reduced translational capacity and slower growth rates that result from either inappropriate tRNA modification or loss of RP function seem to generally improve the resistance against TM-induced ER stress. This possibly indicates that basal ER stress is already reduced due to the translational inefficiency and a concomitant reduction of protein traffic within the ER. In support of this assumption, it has already been demonstrated that translational inhibition by cycloheximide can induce partial TM resistance [45] and that different tRNA modification mutants indeed exhibit protein secretion defects [46].

Our results additionally argue against an important role of the canonical UPR pathway in the observed resistance against ER stress of tRNA modification mutants, since the latter goes along with a clear UPR defect rather than induction of the pathway. Most interestingly, TM resistance observed in several RP deficient mutant strains was at least partially independent of the presence of *HAC1* and therefore occurs without participation of UPR induction [45]. Since our results revealed TM resistance in tRNA modification mutants with chronically reduced mature *HAC1*, we aimed to clarify whether both events are functionally linked or might occur independently of each other.

To this end we introduced an *IRE1* deletion into the *elp3 deg1* double mutant displaying the strongest TM resistance and subsequently scored changes in TM sensitivity by comparing WT, *ire1*, *elp3 deg1* and *elp3 deg1 ire1* mutants. Ire1 represents the endonuclease essential for *HAC1* splicing [26,27,28,29], and therefore, its removal from *elp3 deg1* and WT backgrounds allows us to clarify the role of *HAC1* splicing in resistance against TM-induced ER stress in these mutants. As expected, the absence of Ire1 alone increased TM sensitivity in WT cells but did not alter the TM resistance of the *elp3 deg1* strain, indicating that resistance against ER stress in tRNA modification mutants occurs largely independent of UPR (Figure 3c). We assume that interference with codon translation rates in yeast induces protein homeostasis defects mainly involving cytosolic proteins (Table S1), but at the same time decreases ER stress, resulting in a higher tolerance against ER stress-causing agents and decreased activation of the UPR. This may represent a distinct response as compared to the described effect of reduced codon translation rates in neuronal progenitor cells in mice where increased ER stress and UPR induction were observed [17]. Hence, such differences must be considered when yeast is utilized as a model system for the study of cellular effects of tRNA modification defects.

## Figures and Tables

**Table 1:** *Saccharomycescerevisiae* strains used in this study.

Strain	Genotype	References/Sources
BY4741	*MAT***a**, *his3Δ, leu2Δ, met15Δ, ura3Δ*	Euroscarf, Frankfurt
AB43	BY4741 *ire1Δ::SpHIS5*	This study
RK520	BY4741 *ncs2Δ::SpHIS5 elp6Δ::KanMX4*	This study
RK206	BY4741 *urm1Δ::KanMX4 deg1Δ::SpHIS5*	[16]
RK220	BY4741 *elp3Δ::KanMX4 deg1Δ::SpHIS5*	[16]
AB97	BY4741 *elp3Δ::KanMX4 deg1Δ::SpHIS5 ire1Δ::KlURA3*	This study

**Table 2:** Oligonucleotides used in this study.

Oligonucleotide	Sequence (5′–3′)	Target	References/Sources
Ire1_KO_Fwd	CATTAAAAAAACAGCATATCTGAGGAATTAATATTTTAGCACTTTGAAAACAGCTGAAGCTTCGTACGC	pUG27, pUG72 /*IRE1*	This study
Ire1_KO_Rev	TAACATTAATGCAATAATCAACCAAGAAGAAGCAGAGGGGCATGAACATGGCATAGGCCACTAGTGGATCTG	pUG27, pUG72 /*IRE1*	This study
Ire1_KO+_Fwd	CTTCGGGCAATACCTTCGACT	*IRE1*	This study
Ire1_KO+_Rev	CAACCAAGAAGAAGCAGAGGG	*IRE1*	This study
KO_NCS2_FW	TGCTATTGTCCATCCCTATCCTAGTTTTAAAAATATAATTCTATCAAGTTCAGCTGAAGCTTCGTACGC	pUG27 /*NCS2*	This study
KO_NCS2_RV	TAAATAAATAAATACATAACCATTGGAATAGCGAAGCCTTTGACATTTCAGCATAGGCCACTAGTGGATCTG	pUG27 /*NCS2*	This study
N_NCS2_FW	ACCGATGAGATGAGTGAGAC	*NCS2*	This study
pUG27/SpHIS rev	GTCCAAAGCGATGGCAACGC	*SpHIS5*	This study
HAC1_qPCR_Fwd	GACGACGCTACCTGCCG	*HAC1^i^*	This study
HAC1_qPCR_Rev	ACTGCGCTTCTGGATTACG	*HAC1^i^*	This study
qPCR_ACT1_FW	TTCCAGCCTTCTACGTTTCC	*ACT1*	[16]
qPCR_ACT1_RV	AATCTCTACCGGCCAAATCG	*ACT1*	[16]
HAC1F	CTGGCTGACCACGAAGACGC	*HAC1^u/i^*	[34]
HAC1R	TTGTCTTCATGAAGTGATGA	*HAC1^u/i^*	[34]

## Supplementary Materials

The following are available online at http://www.mdpi.com/2073-4425/9/11/516/s1, Table S1: Gene ontology analysis of protein aggregates identified by mass spectrometry from tRNA modification mutant *ncs2 elp6* ^§^. ^§^ Cell compartment assignment of proteins identified in aggregates from modification mutant *ncs2 elp6* by quantitative mass spectrometry [2] was analyzed with the gene ontology (GO) term function [47] of *Saccharomyces* genome database (SGD) [48].
